# Patient Behavioral Analysis with Smart Healthcare and IoT

**DOI:** 10.1155/2021/4028761

**Published:** 2021-11-03

**Authors:** Anurag Tiwari, Viney Dhiman, Mohamed A. M. Iesa, Haider Alsarhan, Abolfazl Mehbodniya, Mohammad Shabaz

**Affiliations:** ^1^Babu Banarasi Das Institute of Technology & Management, Lucknow, India; ^2^Department of Cardiology, PGIMER, Chandigarh, India; ^3^Department of Physiology, Al Qunfudah Medical College, Umm Al Qura University, Mecca, Saudi Arabia; ^4^Mustansiriyah University, College of Medicine, Baghdad, Iraq; ^5^Department of Electronics and Communications Engineering, Kuwait College of Science and Technology (KCST), Doha Area, 7th Ring Road, Kuwait; ^6^Arba Minch University, Ethiopia; ^7^Department of Computer Science Engineering, Chandigarh University, Punjab, India

## Abstract

Patient behavioral analysis is the key factor for providing treatment to patients who may suffer from various difficulties including neurological disease, head trauma, and mental disease. Analyzing the patient's behavior helps in determining the root cause of the disease. In traditional healthcare, patient behavioral analysis has lots of challenges that were much more difficult. The patient behavior can be easily analyzed with the development of smart healthcare. Information technology plays a key role in understanding the concept of smart healthcare. A new generation of information technologies including IoT and cloud computing is used for changing the traditional healthcare system in all ways. Using Internet of Things in the healthcare institution enhances the effectiveness as well as makes it more personalized and convenient to the patients. The first thing that will be discussed in the article is the technologies that have been used to support the smart class, and further, there will be a discussion on the existing problems with the smart healthcare system and how these problems can be solved. This study can provide essential information about the role of smart healthcare and IoT in maintaining behavior of patent. Various biomarkers are maintained properly with the help of these technologies. This study can provide effective information about importance of smart health system. This smart healthcare is conducted with the involvement of proper architecture. This is treated as effective energy efficiency architecture. Artificial intelligence is used increasingly in healthcare to maintain diagnosis and other important factors of healthcare. This application is also used to maintain patient engagement, which is also included in this study. Major hardware components are also included in this technology such as CO sensor and CO_2_ sensor.

## 1. Introduction

This article is about the importance of smart healthcare inpatient behavioral analysis. In the digital world, everything is getting transformed and becoming informative. The advancement and the rapid growth of technology allow traditional biotechnology as well as medicine to get digital and become more informed. New information technology has emerged by the incorporation of smart healthcare. The smart care health system is a multilevel change. The changes involved in the smart healthcare system for analyzing the patient behavioral include disease-centered to patient-centered, changed concept of management and prevention, and changing of information construction to the regional medical transformation to clinical informatization.

**Figure 1:**
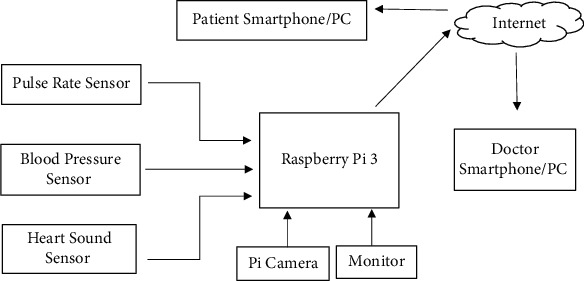
Block diagram.

These changes are based on the current needs of the patients by improving the efficiency and performance of the healthcare system. The implementation of IoT will help in developing a smart healthcare system. The challenges in the traditional medical system will be overcome through the smart healthcare system. In this article, the concept of smart healthcare will be discussed as well and the features and challenges of smart healthcare have been also discussed. The key technology is IoT that is used in the smart healthcare system, and it will be also discussed, and how effectively it can analyze the patient behavior will be discussed in the article. IoT is implemented successfully to maintain smart health monitoring system, which is represented in block diagram. Raspberry Pi 3 is used to maintain pulse rate sensor, blood pressure sensor, and heart sound sensor.  Figure 1 shows the block diagram for the same, and Table 1 shows the list of future and available sensors and their applications for detecting patient behavioral analysis.

### 1.1. Literature Review

Internet of Things has a major contribution in the healthcare sector and improves the lives of various people throughout the world. From the literature review, it has been cleared that using IoT in the healthcare institution can enhance the quality of the medical facilities as well as enhance the efficiency of healthcare management [1]. This article is focusing on the challenges and difficulties faced by people in treatment as well as how a smart healthcare system allows doctors to track the disease and provide digital medicine to their patients [2]. Cloud computing, artificial intelligence, big data, and soft computing are the advanced technologies of the IoT that are used for monitoring patients' health and blood pressure as well as body temperature. Electrocardiograms, monitoring, glucose level sensing, and emergency healthcare as well as wheelchair management are connected through these devices. This facilitates the doctor to check the patient's status from any corner of the world. The article will highlight the automated wheelchair, warfarin, wearables, wireless transmission, and receivers [3].

### 1.2. Importance of Smart Healthcare System

Healthcare system consists of multiple participants including hospitals, patients, doctors, and research organizations. The cornerstone of smart healthcare consists of various information technologies that include IoT, cloud computing, mobile Internet, 5G, artificial intelligence, and big data together with biotechnology [4]. Smart healthcare uses these technologies for the healthcare system. Wearable gadgets are used by patients with wearable gadgets to keep track of their health every time, provide medical care by virtual helpers, and use remote places to implement remote services; a variety of sophisticated clinical decisions are employed by doctors to support systems to improve diagnosis. According to Baker et al., integrated information platforms are handled by doctor's form that incorporates tools like picture archiving, communication systems and information management system of laboratory, and the electronic medical record [5]. Surgical robots and mixed reality technology can help with more precise surgery.

People materials, as well as the supply chain of the hospitals, are maintained through radiofrequency identification (RFID), with integrated management platforms, collecting data and assisting decision-making. Mobile medical platforms are widely used for improving mobile medical platforms [6]. At the scientific research institutes, instead of manual drug screening, machine learning is widely used as well as big data may be used to discover suitable participants. The cost and risk of medical procedures can be effectively reduced with the help of smart healthcare. The utilization efficiency of medical resources as well as self-service medical care as well as promotion of regional exchange and cooperation provides personalized medical services by implementing IoT in the smart healthcare system [7].

### 1.3. The Architecture of the Smart Healthcare System

The smart healthcare institute that has been developed in this article is capable of making decisions according to the observed conditions of the patient's pulse rate and heartbeat as well as body temperature. The given architecture is also an energy-efficient solution as it does not turn on the senior management every time. The sensors and the cost and lifetime of the system are handled through the used algorithm in the architecture. The issues faced by the remote monitoring of the patients help in providing them a necessary treatment through expert doctors in the hospital. Communication channels and embedded internal as well as external sensors consist in the monitoring of the smart healthcare monitoring system as well as patient management system. Different levels of refinement are used for performing these activities including the management layer, device layer, and network layer. The working performance of telemedicine in rural areas gets improved through telemedicine. IoT has helped in implementing the device in a rural clinic. All data of the patients are taken by the device then sent to the doctor concerned in the hospital. These data get analyzed by the doctors and suggest some necessary steps for the patient best treatment.

### 1.4. Diagnosis and Treatment Based on Artificial Intelligence

Various healthcare institutions are overcome by the challenges of AI implementation. Rule-based system includes NHS11 and lacks the accuracy of more algorithm; machine learning-based systems are embedded in the EHR system that is used. Maintenance of these clinical decision support systems is quite challenging due to the evolvement of medical knowledge. The knowledge generated through metabolic and other genomic proteomic approaches is difficult to cope by the support system. Compared to clinical practice, they are more used in research labs as well as by tech companies. These clinical decision support systems are difficult to maintain as medical knowledge evolves, and they are unable to cope with the sources of data and knowledge generated by metabolic and other genomic proteomic approaches to care. It is more prevalent in research labs and tech companies than in clinical practice to analyze the behavior of patients [8].

### 1.5. Patient Engagement and Adherence Applications

Adherence and involvement of the patient are considered a last mile challenge for the healthcare industry. It acts as a challenge between the good health results. The participation of the patients increased through the better outcome. Better outcomes will also lead to good utilization as well as provide better member experience and financial results. These issues can be addressed through big data and AI. Hospitals and providers used clinical experience for establishing a care plan for the patients that helps in knowing the improvement of acute or chronic patients [9, 10]. That does not matter if the patient does not make the necessary behavioral changes, such as arranging a follow-up visit, losing weight, filling medicines, or adhering to a treatment plan. When a patient fails to follow a treatment plan or take prescription medications as directed, this is a big issue.

### 1.6. IoT-Enabled Healthcare

Highly creative linked health technolines are driven by the positioning of the healthcare business that includes services of IoT and IoT apps, as well as solutions. Improvisation of healthcare services is the main objective of digital health by reducing the cost [11]. In smart healthcare, all remote patients are allowed to get diagnosis and treatment by using wearable devices. The patient's status is analyzed with the help of devices that are embedded with the sensor. Wearable devices collect the heart rate and blood glucose as well as oxygen saturation and transfer them to the caregiver through the smartphone of the patients [12]. Telehealth allows doctors to consult with patients without having to travel to a clinic or hospital. Behavior modification is a technique that can assist patients in changing poor behaviors and adopting healthier lifestyles to better control their health.  Table 2 below describes a few healthcare case studies.

## 2. Materials and Methods

The materials and method used in the article for analyzing the behaviour of patients with smart healthcare are based on the advancement of the technology. A comprehensive survey has been done on IoT-enabled healthcare. IoT-enabled healthcare involves IoT healthcare network (IoThNet) that is the most important element in the smart healthcare system [13]. IoThNet consist of IoThNet platform, IoThNet architecture, and IoThNet topology. In various services and applications, IoT-enabled healthcare gets divided, and each service gets one potential vertical. More than 300 clinical leaders and healthcare staffs were present. More than 70% of the respondents claimed that less than half of their patients were highly involved, and 42% stated that less than a quarter of their patients were extremely in an IoT network; a health management system can observe a patient's basic symptoms such as oxygen saturation percentage, heart rate, and body temperature [14].

SpO_2_, heartbeat, eye blink, and temperature sensors were provided as capturing elements, while an Arduino UNO was used as a processing device. The system was sent; there are no defined performances for patients. In an IoT ecosystem, a healthcare monitoring kit, heartbeat, body temperature, ECG, and respiration were among the basic characteristics determined by the designed system. Temperature sensor, blood pressure sensor, ECG sensor, pulse sensor, and Raspberry Pi are the hardware components used here [15]. The data was gathered from the sensors and transferred to a Raspberry Pi to process before being delivered back to the IoT network.

The system's main flaw is that no data visualization interfaces have been created. In the survey, as a noninvasive method, a pulse rate detection system was used. A system was proposed that utilized the plethysmography method and digitally presented the results, making it a real-time monitoring gadget. In comparison to other intrusive treatments, the procedure has proven to be safe for the patient. The finger blood flow can be tracked through mobile light and cameras for calculating the blood flow-based cardiac output [16]. Integrated gadgets are developed through the developed system that was sent to the computer through the person's pulse. It allows users to check the heart rate by just looking in the phone not thoroughly by the person's hand. This is an awesome way; however, it requires constant heart monitoring that completely works with the cardiovascular disease sensing system for the smartphone and has the goal of discovering the tool that is created through the given time and phones [17].

The prototype produced only captured coronary rhythm, but not heart rate, and failed to detect any cardiovascular disease. Arduino-based health parameter surveillance framework managed through a mobile device Arduino Uno gets delivery of the captured analog sensor data board. The captured values of analog get transformed into digital information through inbuilt analog to digital converter. The physical properties were exchanged with the designed device through Bluetooth [18]. The Bluetooth gadget had an area that did not cover a large area. An IoT safety monitoring device that adapts the framework's setup is divided into three layers: the control layer, the transport layer, and the device layer. In the control section, a pulse sensor was employed to detect a pulse and DS18B20 sensor was utilized to measure. The data from the Arduino into the cloud gets loaded with the help of Wi-Fi module and Ethernet shield on the transport layer. However, because an Arduino Uno was utilized, several sensors were unable to be appropriately processed [19]. To track smart houses and heartbeats, a wireless sensor network (WSN) was created. There is a parallel data processing; Spartan3 is used with FPGA architecture. On the LCD, results of the MCU are shown and through the microcontroller, the sensors get linked.

## 3. Major Hardware Components

Various hardware has been used in the smart healthcare system. Body temperature sensor, ESP32 processor, room-temperature sensor, heartbeat sensor, CO sensor, and CO_2_ sensor have been used for developing the smart care system for healthcare. The I2C/UART and SPI/SDIO interfaces on the EPS32 allow it to have communication with other Wi-Fi and Bluetooth devices. The sensitivity of the sensor can be adjusted using the potentiometer. The detection and quantification of NH_3_, smoke, nicotine, benzene, and CO_2_ are done through the MQ-135 gas sensors used in the air quality control. The MQ-135 sensor module has a digital pin that allows it to work without the use of a microcontroller, which is useful for detecting specific gases. The analog pins are used to determine the gases in PPM.

**Figure 2:**
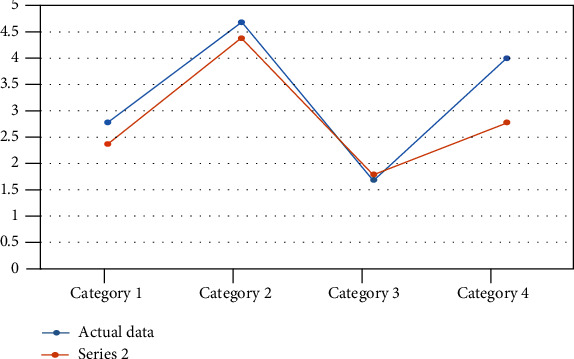
Deviation of data in room humidity.

The plethysmography principle was used to design the heartbeat sensor. The changing in the blood volume can be easily detected by it in any organ that is responsible for the intensity of light to travel through that organ. The pulse timings are even more important in systems that track the heart rate [20] .When light is devoured by the blood, the rate of heartbeat affects the blood volume distribution, and the signal pulses are equivalent to the heartbeat pulses. ESP32 is used as an IoT tool that offers a Linux system at a cheap price. GPIO pins are used by ESP32 for connecting actuators to the device sensors [21]. Acquisition of IoT with ESP32 brings new creativity to the healthcare system. With integrated antenna switches, control amplification, RF balloon, low-noise amplifier, and filters, as well as power management modules, the ESP32 is particularly well designed. It can work as a slave to a host MCU or a stand-alone scheme, reducing the amount of time spent interacting with the main application processor [22]. Compared to Kelvin's linear temperature sensors, LM35 has more advantages in that realistic centigrade scaling prevents the consumer from deleting the great constant voltage from the display. MQ-9 is suitable for detecting LPG, CO, and CH4 [23]. Measurements can be taken quickly because of their high sensitivity and quick reaction time [24]. Researchers are also providing security protocols for exchanging information among clients and servers and to maintain the confidentiality of the shared information as in Figure 2 [25].

## 4. Discussion and Results

A smart healthcare system allows doctors to analyze the patient's behaviour that helps in providing a proper diagnosis to them [26]. Monitoring of the smart health management system allows patients to check their health status according to their comfort at any place [27]. Smart healthcare systems allow by providing this status on a digital platform that can be visible from any corner of the world. The real-time value of the health system is allowed by the smart healthcare system as well as it shows how it can be implemented in the real-life world [28]. The disease of the patients can be easily identified with the smart healthcare system, and diagnosis can be easily made by observing the various symptoms. The smart healthcare system allows the doctors to use the log of the patient's body conditions for analyzing the medicine effect on other things.

The devised methodology was put to the test with a variety of participants of varying ages in a variety of situations. The real value and observed value have been manually determined from the built system in the test scenarios for a body temperature, heartbeat, and room temperature sensors [29]. Room temperature sensor is only used for measuring the humidity; in this case, the error rate based on the data has been calculated to demonstrate the system's effectiveness. The data of MQ-9 and MQ-135 on the web server has been observed because there are no other means to measure the harmful gas level [30]. In Tables 3, 4, and 5 given below, the body temperature and room temperature, as well as the error rate for heartbeat, have been provided [31].

**Figure 3:**
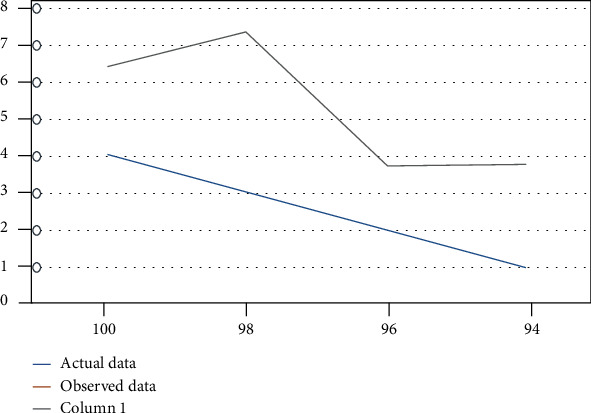
Data collected for measuring the body temperature of patients.

**Figure 4:**
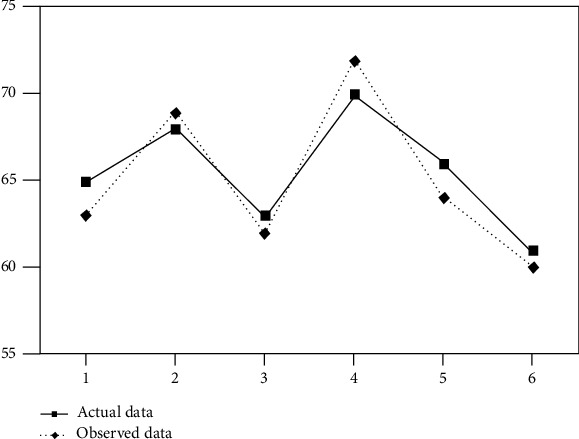
Data collected for room humidity.

**Figure 5:**
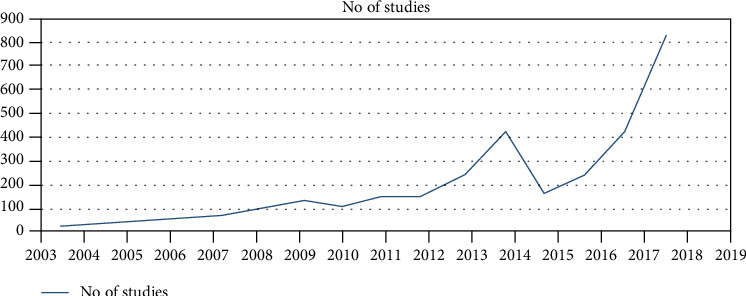
No. of studies regarding smart healthcare.

**Figure 6:**
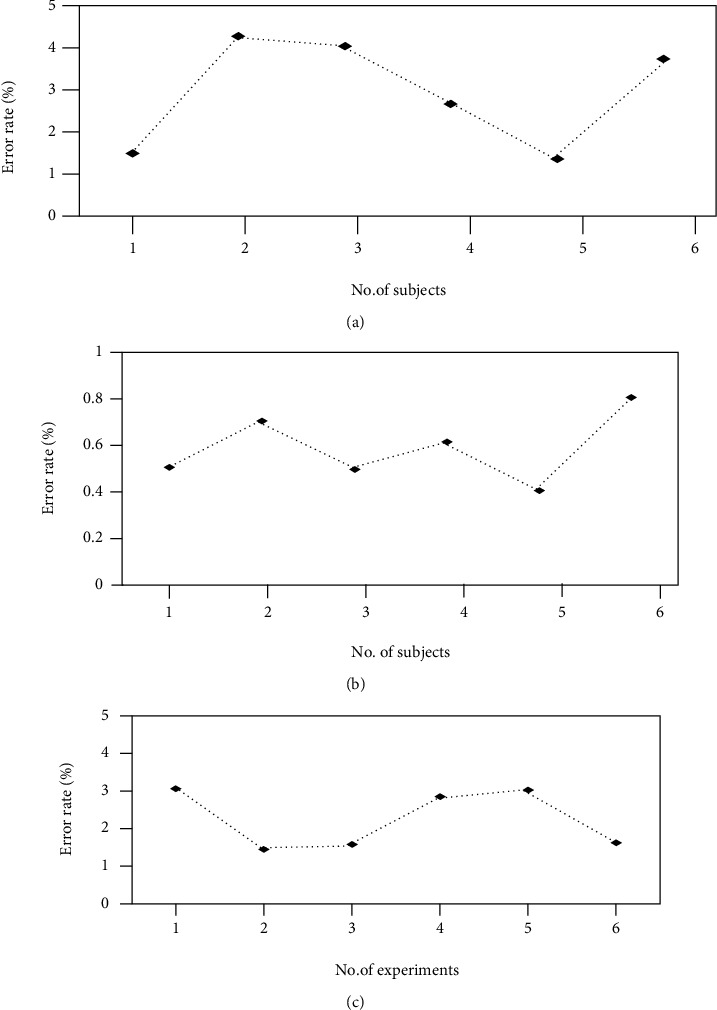
(a–c) Error rate for a developed system for (a) heart rate, (b) body temperature, and (c) room humidity.

In Figure 3, the deviation of the data has been obtained through the developed system. A clear deviation can be shown between actual and observed data showing the heart rate data that has been collected from various patients of different ages in healthcare. The movement that is caused by the patients during the treatment was responsible for causing the deviation. There is a chance of error due to motion artifacts that create deviation. This can lead to inaccurate data. Light scattering from other sources is also responsible for causing deviation. The mispositioning of the system is responsible for the deviation of the body temperature. PPM units are used for measuring the toxic gases including CO and CO_2_ levels. The room condition as well as the specific patient can be monitored by the medical staff. The data can be accessed through the password only, which protects the data of the patients. The system can be monitored only by the authorized staff. From the remote location, the doctor can easily monitor the status of the patient. The level has been specified for the threshold for these measurements. The medical staff is allowed to take necessary steps for healthcare in the case when the data crosses its level [32]. In Figure 4, the developed system for the room humidity measurement, body temperature, heart rate, and error rate has been found, which has been shown in Figure 5 and Figures 6(a)–6(c). In the cases, the highest rate of error has been found about 4.28%, 3.07%, and 0.81%. An error rate of more than 5% is not acceptable in all cases.

## 5. Conclusion

Based on this study, it is concluded that the use of Internet of Things can play a crucial role in maintaining the development of smart healthcare. The use of analog machine in maintaining body temperature is evaluated properly in this study. Actuarial machine is also used to maintain body temperature of patients, which is concluded in this study. Flexibility of healthcare system is improved with the help of Internet of Things. To provide proper diagnosis towards patients, it is important to implement Internet of Things. Based on this study, it is noticed that proper architecture is present in maintaining growth and development of smart healthcare system. It has been concluded that smart healthcare has key contribution in analyzing the patient's behavior. Better health self-management can be enjoyed by the patients by smart healthcare. As compared to the traditional healthcare, smart healthcare system can reduce personnel pressure, relieve costs, and improve the patient's medical experience. The status of medical resources can be enhanced with the help of smart healthcare. Implementation of IoT in the development of smart healthcare allows the healthcare staff to easily detect the types of diseases as well as the diagnosis can be done properly in smart healthcare systems as compared to traditional medical facilities. Results shows that the developed system monitors the room humidity measurement, body temperature, and heart rate; also, error rate has been found. The highest rate of error has been found about 4.28%, 3.07%, and 0.81%, respectively. An error rate of more than 5% is not acceptable in all cases. With several advantages, some problems in the smart healthcare system still exist that can be overcome with the development of the technologies as well as with joint effort of the doctors, healthcare staff, and patients as well as medical institutes.

## Figures and Tables

**Table 1:** List of future and available sensors and their applications for detecting patient behavioral analysis.

Biomarker	CVD	COPD	PD/HD	Diabetes
Respiratory rate	True	True	True	?
ECG	True	True	True	True
Surface EMG	True	True	True	/?
Gait (posture)	True	True	True	?
Skin temperature	True	True	True	True
Blood pressure	True	True	True	True
Oxygenation	True	True	?	?
Heart sound	True	True	?	?
Title volume	True	True	True	?

**Table 2:** Healthcare case studies with the description.

Parameter	Company	Description
Remote patient monitoring	Vivity	Monitor and management of remote heart failure
	CardioMEMS	The implantable device is used for monitoring the remote heart failure monitoring as well as management
	AliveCor	Arrhythmia diagnosis and monitoring through ECG
	Dexcom	Glucose monitoring continuously, linked to a smartphone app and social media network

Behavior modification	Omada	Preventing from diabetes by providing weight loss coaching

Telehealth	Doctor on demand	The meeting is conducted between patients and doctor through virtual mode

**Table 3:** Smart healthcare monitoring system using IoT.

Subjects	Actual data (bpm)	Observed data (bpm)	Error (%)
S1	65	67	1.47
S2	69	72	4.25
S3	75	76	4.03
S4	74	74	2.63
S5	72	71	1.32
S6	79	80	3.70

**Table 4:** Using of the analog machine (actual) for collecting data for body temperature.

Subjects	Actual data (*F* degree)	Observed data (*F*)	Error (%)
S1	96.3	98.7	0.50
S2	97.4	98.7	0.70
S3	98.2	97.6	0.50
S4	96.7	97.1	0.61
S5	97.4	97.0	0.40
S6	98.1	97.5	0.79

**Table 5:** Collection of room humidity through the actuarial machine and the observed system.

Experiments	Actual data (%)	Observed data (%)	Error (%)
1	64	62	3.05
2	67	68	1.45
3	62	61	1.56
4	69	70	2.80
5	65	63	3.01
6	60	59	1.59

## Data Availability

The data captured to support the findings of this study are available from the corresponding author upon request.
